# Resistance to Dihydroartemisinin

**DOI:** 10.3201/eid1211.060903

**Published:** 2006-11

**Authors:** Sandrine Cojean, Véronique Hubert, Jacques Le Bras, Rémy Durand

**Affiliations:** * Hôpital Bichat Claude Bernard, Assistance Publique–Hôpitaux de Paris, Paris, France;; † Université Paris 5, Paris, France;; ‡ Hôpital Avicenne, Bobigny, France;; § Université Paris 13, Bobigny, France

**Keywords:** Plasmodium falciparum, artemisinin derivatives, drug resistance, letter

To the Editor: The emergence of widespread resistance to chloroquine and sulfadoxine-pyrimethamine in Africa has caused a sharp rise in deaths from malaria. The World Health Organization therefore urgently recommends replacement of these drugs, particularly with combinations that include an artemisinin compound (AC) (*1*). In 2006, although >40 countries have adopted artemisinin-based combination therapies as their first-line treatment for malaria, only a few of these countries actually use these combination therapies because of limiting factors such as high cost (*2*). When used as monotherapy, ACs are associated with high rates of recrudescence, possibly because of their short elimination half-lives (*3*). Most artemisinin-based combination therapies contain, in addition to ACs, a partner drug against which resistance has already developed (e.g., mefloquine, amodiaquine, lumefantrine); reports of relatively low efficacy of the combination artesunate-amodiaquine have been recently published (*4*). In 2005, Jambou et al. claimed to have found the first cases of in vitro Plasmodium falciparum resistance to ACs (*5*).

We assessed the in vitro susceptibility to dihydroartemisinin (dhART), the biologically active metabolite of artemisinin derivatives, of P. falciparum isolates from travelers returning to France from various African countries during 2004–2006. In addition, we searched for polymorphism in the P. falciparum adenosine triphosphatase-6 (PfATPase6) gene, which was reported to be associated with in vitro artemether resistance (*5*). We also studied polymorphism (a 3-bp indel) in the gene of the ABC transporter G7, which was reported in 2005 to be associated with in vitro response to artesunate (*6*).

Determination of in vitro dhART susceptibility by using the isotopic semimicrotest method (*7*) was successful for 397 isolates. The most represented countries were Cameroon (17%), Cotê d'Ivoire(14.5%), Mali (12%), Comoros Islands (8.5%), and Senegal (6.5%). Patients were <75 years of age (mean 31, SD 17 years), and the male:female ratio was 1.5:1. The 50% inhibitory concentration (IC_50_) values ranged from 0.02 to 31.8 nmol/L, with a geometric mean of 1.31 nmol/L and a median of 0.68 nmol/L. IC_50_ values were <1 nmol/L for 264 isolates, 1–10 nmol/L for 127, and >10 nmol/L for 6. Thus, some isolates showed a diminished susceptibility to dhART, but only 1 isolate had an IC_50_ >30 nmol/L (31.8 nmol/L).

DNA sequencing of 900-bp and 240-bp PCR products, including the 769 and the 243/263 PfATPase6 codons, respectively, was performed in a subsample of 154 isolates. All isolates had the S769 wild codon except 1 susceptible isolate (IC_50_ = 0.83 nmol/L), which had a S769N mutant type codon (Table). We found no polymorphism in codon 263. This position may be scrutinized to monitor anticipated artemisinin resistance, according to a recently published structure-function study (*8*). Conversely, we found 2 isolates that had IC_50_ values of 4.2 nmol/L and 6.4 nmol/L and that showed an H243Y mutant type codon. The role of such a polymorphism appears unclear. We found no association between the 3-bp indel in G7 and in vitro dhART susceptibility because mutants were regularly distributed in highly susceptible isolates and in isolates having a diminished susceptibility.

**Table:** Polymorphism in *PfATPase6* and G7 genes and in vitro susceptibility to dihydroartemisinin of 154 *Plasmodium falciparum* isolates*

Gene	Predicted products	Position	Amino acid	Nucleotide change	No. isolates	Dihydroartemisinin IC_50_ (nmol/L)
*ATPase6*	Sarcoplasmic reticulum calcium- transporting ATPases	769	S	AGT	153	0.1–31.8
			S→N	AAT	1	0.83
		263	L	TTA	154	0.1–31.8
			L→S	TCA	0	
		243	H	CAT	152	0.1–31.8
			H→Y	TAT	2	4.2; 6.4
G7	ABC transporter	1,390	Wild	(AAT)_4_	69	0.1–25.9
			Mutant (3-bp indel)	(AAT)_3_	85	0.15–31.8

*PfATPase, *Plasmodium falciparum* adenosine triphosphatase; IC_50_, 50% inhibitory concentration.

For our samples obtained during 2004–2006, the geometric mean IC_50_ value for dhART was very close to values found in Cameroon during 1997–1998 (mean dhART IC_50_ = 1.11 nmol/L) (*9*), in Senegal in 2001 (mean artemether IC_50_ = 1.3 nmol/L) (*5*), and in Republic of Congo during 2005–2006 (mean dhART IC_50_ = 1.02 nmol/L) (*10*). Ringwald et al. observed a narrower range of IC_50_s, but their series included only 65 samples (*9*). Previous comparisons between ACs suggested that dhART is 1.7 times more potent than artemether against P. falciparum (*9*). Thus, the highest IC_50_ value for artemether observed by Jambou et al. in Senegal (44.7 nmol/L) (*5*) is comparable to the highest IC_50_ value for dhART in our series (31.8 nmol/L). The resistance levels of ACs are still undefined. For artemether, Jambou et al. used a threshold of 30 nmol/L to evaluate the association between the S769N mutation and in vitro susceptibility. The presence of ATPase6 S769N was not associated with diminished in vitro susceptibility in our series. Conversely, the only S769N mutant that we observed was found in a fully susceptible isolate. Thus, we confirmed that polymorphism exists in this gene in positions 769 and 243, but we did not prove an association between these point mutations and resistance to ACs. Similarly, our results did not support the hypothesis of an association between the 3-bp indel in G7 and resistance to ACs.

ACs, considered the most important class of antimalarial drugs, merit close surveillance for susceptibility. Continued monitoring of the efficacy of their associated partner drugs also appears to be essential.

## References

[1] Attaran A, Barnes KI, Curtis C, D'Alessandro U, Fanello C, Galinski MR, WHO, the Global Fund, and medical malpractice in malaria treatment. Lancet. 2004;363:237–40. 10.1016/S0140-6736(03)15330-5 14738799

[2] Mutabingwa TK. Artemisinin-based combination therapies (ACTs): best hope for malaria treatment but inaccessible to the needy! Acta Trop. 2005;95:305–15. 10.1016/j.actatropica.2005.06.009 16098946

[3] Ittarat W, Pickard AL, Rattanasinganchan P, Wilairatana P, Looareesuwan S, Emery K, Recrudescence in artesunate-treated patients with falciparum malaria is dependent on parasite burden not on parasite factors. Am J Trop Med Hyg. 2003;68:147–52. 12641403

[4] Grandesso F, Hagerman A, Kamara S, Lam E, Checchi F, Balkan S, Low efficacy of the combination artesunate plus amodiaquine for uncomplicated falciparum malaria among children under 5 in Kailahun, Sierra Leone. Trop Med Int Health. 2006;11:1017–21. 10.1111/j.1365-3156.2006.01655.x 16827702

[5] Jambou R, Legrand E, Niang M, Khim N, Lim P, Volney B, Resistance of *Plasmodium falciparum* field isolates to in-vitro artemether and point mutations of the SERCA-type PfATPase6. Lancet. 2005;366:1960–3. 10.1016/S0140-6736(05)67787-2 16325698

[6] Anderson TJC, Nair S, Qin H, Singlam S, Brockman A, Paiphun L, Are transporter genes other than the chloroquine resistance locus (*pfcrt*) and multidrug resistance gene (*pfmdr*) associated with antimalarial drug resistance? Antimicrob Agents Chemother. 2005;49:2180–8. 10.1128/AAC.49.6.2180-2188.2005 15917511 PMC1140548

[7] Le Bras J, Andrieu B, Hatin I, Savel J, Coulaud JP. *Plasmodium falciparum*: interpretation of the semi-microtest of in vitro chemosensitivity by H3-hypoxanthine incorporation [in French]. Pathol Biol (Paris). 1984;32:463–6. 6377209

[8] Uhlemann AC, Cameron A, Eckstein-Ludwig U, Fischbarg J, Iserovich P, Zuniga FA, A single amino acid residue can determine the sensitivity of SERCAs to artemisinins. Nat Struct Mol Biol. 2005;12:628–9. 10.1038/nsmb947 15937493

[9] Ringwald P, Bickii J, Basco LK. In vitro activity of dihydroartemisinin against clinical isolates of *Plasmodium falciparum* in Yaounde, Cameroon. Am J Trop Med Hyg. 1999;61:187–92. 10463665 10.4269/ajtmh.1999.61.187

[10] Pradines B, Hovette P, Fusai T, Atanda HL, Baret E, Cheval P, Prevalence of in vitro resistance to eleven standard or new antimalarial drugs among *Plasmodium falciparum* isolates from Pointe-Noire, Republic of the Congo. J Clin Microbiol. 2006;44:2404–8. 10.1128/JCM.00623-06 16825356 PMC1489504

